# The Association Between Serious Leisure and University Students’ Life Satisfaction: Focusing on the Broaden-and-Build Theory of Positive Emotions

**DOI:** 10.3390/bs15121677

**Published:** 2025-12-03

**Authors:** Kyulee Shin, Sukkyung You, Youngmi Sohn

**Affiliations:** 1 Department of Sports Sciences, Seoul National University of Science & Technology, Seoul 01811, Republic of Korea; kyuleeshin@seoultech.ac.kr; 2 College of Education, Hankuk University of Foreign Studies, Seoul 02450, Republic of Korea; 3 Department of Counseling & Psychotherapy, Konyang University, Nonsan-si 32992, Republic of Korea; shonym@konyang.ac.kr

**Keywords:** serious leisure, positive emotion, positive thought, positive psychological capital (PsyCap), life satisfaction, Fredrickson, broaden and build theory

## Abstract

Positive psychological capital (PsyCap) is closely linked to well-being. This study examined the role of serious leisure in fostering PsyCap among Korean university students, drawing on Fredrickson’s broaden-and-build theory of positive emotion. Using structural equation modeling, we investigated whether serious leisure participation predicts life satisfaction through the sequential mediating effects of positive emotion, positive thought, and PsyCap. A stratified sample of 480 Korean college students completed measures of serious leisure, positive emotion, positive thought, PsyCap, and life satisfaction. The findings revealed significant indirect associations, supporting the applicability of Fredrickson’s broaden-and-build theory in explaining how serious leisure enhances life satisfaction via positive psychological resources. These findings highlight serious leisure as a meaningful pathway to psychological well-being and suggest the value of university-based programs that promote constructive leisure engagement. The key contribution of this study lies in extending the understanding of serious leisure by empirically linking it to PsyCap and life satisfaction in a non-Western context, identifying it as a developmental resource that fosters long-term well-being.

## 1. Introduction

University students face various challenges such as academic pressure, social adjustment, and career uncertainty, which can significantly impact their mental health and overall quality of life (Seligman, 2020). In Korea, these challenges are exacerbated by intense educational competition, high societal expectations for success, and cultural norms that emphasize collectivism and conformity (Hofstede, 2011; S. Lee & Park, 2021). Therefore, enhancing the well-being and life satisfaction of Korean university students is a crucial task. Positive psychology, pioneered by Martin Seligman (Seligman, 1998), provides a framework for leveraging individual strengths to promote well-being. Within this framework, positive psychological capital (PsyCap) and serious leisure have emerged as key constructs associated with improved psychological functioning. However, despite increasing scholarly interest, recent research suggests that the pathways through which leisure experiences influence PsyCap and life satisfaction remain insufficiently understood, particularly in East Asian contexts (Dirzyte et al., 2022). This underscores the need for studies that clarify these mechanisms among Korean university students, whose cultural and educational environments differ markedly from Western settings where most evidence has been generated.

Positive psychological capital (PsyCap) is characterized by four components—self-efficacy, optimism, hope, and resilience—representing an individual’s positive psychological state (Luthans, 2002). Self-efficacy refers to confidence in one’s ability to successfully perform tasks and achieve goals; optimism reflects general expectation for positive outcomes in the future; hope involves persistent effort toward goals and the ability to find alternative paths when faced with obstacles; resilience denotes the capacity to recover from adversity. Together, these components predict enhanced performance, well-being, and life satisfaction (Luthans, 2002; Luthans et al., 2006). These components interact dynamically—e.g., hope correlates with optimism and resilience with self-efficacy—though their interplay is complex and context-dependent (Avey et al., 2011). Positive emotions, such as joy or pride (Watson et al., 1988), strengthen optimism and hope, while positive thoughts—adaptive, goal-oriented cognitive patterns (Ingram & Wisnicki, 1988)—bolster self-efficacy and resilience. Recent empirical work indicate that cultivating positive psychological resources is particularly important in collectivistic cultures, where academic pressure and social expectations can suppress emotional expression and adaptive coping (Jung & Oh, 2018). High PsyCap is associated with life satisfaction and well-being, although full flourishing also requires additional elements such as meaning and social relationships (Luthans & Youssef-Morgan, 2017; Seligman, 2020).

**Figure 1:**
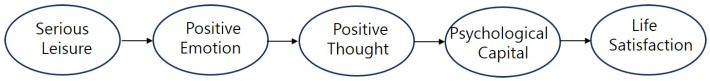
Research model.

These processes are theoretically grounded in Fredrickson’s (2001) broaden-and-build theory, which posits that positive emotions expand an individual’s cognitive and behavioral repertoires, facilitating the construction of enduring psychological assets such as PsyCap (Fredrickson, 2004; Cohn & Fredrickson, 2009; Tugade & Fredrickson, 2004). Prior studies indicate that positive affect promotes psychological flexibility, which may contribute to PsyCap development through sequential pathways (Tugade & Fredrickson, 2004; Fredrickson, 2004). Yet, the recent literature highlights the scarcity of studies empirically testing these sequential mechanisms within structured leisure contexts, especially among non-Western student populations (Dirzyte et al., 2022). Building on this framework, this study examines whether participation in serious leisure is associated with higher levels of positive emotion, which then relate to positive cognition (i.e., positive thought), PsyCap, and ultimately life satisfaction (Figure 1).

Serious leisure, a concept developed by Stebbins beginning in the 1970s, represents the systematic pursuit of freely chosen activities through which participants cultivate specialized skills, knowledge, and identities over time. Rather than implying externally imposed “structure,” Stebbins (1982) emphasizes that serious leisure entails a *systematic* and personally meaningful commitment—distinguishing it from the spontaneous, episodic nature of casual leisure. The six defining qualities of serious leisure—perseverance, career-like progression, significant personal effort, durable benefits, unique ethos and social world, and strong identification and commitment—together highlight its developmental potential. These features connect serious leisure to broader life processes, including the formation of central life interests, identity development, and community contribution. Participation often involves entry into affinity-based social worlds that foster group identity and belonging, supporting students’ navigation of liminal transitions (e.g., entering university or preparing for the workforce), consistent with Turner’s notions of liminality and communitas (Turner, 1969).

Since its emergence, the Serious Leisure Perspective (SLP) has developed into one of the most influential frameworks in leisure sociology. Early work on amateurism (1973–1981) laid the groundwork for the notion of “durable benefits,” such as self-actualization, self-expression, regeneration, social attraction, group accomplishment, contribution, enhanced self-image, and lasting satisfaction—core components that highlighted the long-term personal and social rewards of sustained leisure participation. The SLP gained firmer theoretical shape in 1982, when Stebbins articulated six defining qualities of serious leisure and reframed leisure not as simple recreation but as a systematic, career-like pursuit. Throughout the 1990s, the framework expanded through the classification of amateurs, hobbyists, and volunteers, as well as the introduction of casual leisure as a contrasting form. Stebbins’s work during this period also established the concept of a “leisure career,” underscoring the developmental trajectory of long-term participation. In the 2000s, the SLP was further refined with the articulation of five leisure career stages (Stebbins, 2001), the introduction of project-based leisure (Stebbins, 2005), and the development of the Serious Leisure Inventory and Measure (SLIM), which enabled more robust empirical evaluation (Gould et al., 2008). More recent scholarship has integrated the three forms of leisure (serious, casual, and project-based) (Elkington & Stebbins, 2014) and emphasized broader social implications—such as educational outcomes (Heo et al., 2013a), community engagement (K. J. Lee et al., 2018), aging (Brown et al., 2008), and even potential risks such as overinvestment or addiction (Veal, 2017)—demonstrating both the versatility and the boundaries of the framework.

Across its 40+ year evolution, the SLP has been applied to a wide array of topical arenas, including sports (Lower-Hoppe et al., 2023), arts (Stalp et al., 2023), volunteering (Wang, 2021), tourism (Cai et al., 2020), environmental stewardship (Heidari & Gravelle, 2025), and educational enrichment (Mouratidou et al., 2024). Its practical utility lies in explaining how individuals cultivate identity, expertise, and social capital through sustained pursuit of meaningful activities (Lamont et al., 2014). Theoretically, it illuminates the intersection between leisure participation, personal development, community membership, and the formation of central life interests (Elkington & Stebbins, 2014). At the same time, scholars have noted several limitations: a tendency to understate structural constraints (e.g., income, time, social disapproval) (Hubbard & Mannell, 2001), potential overemphasis on individual agency (Rojek, 2010), and limited attention to cultural variation in leisure careers. These limitations are particularly relevant in Korea, where academic competition, parental expectations, and time scarcity may restrict opportunities for sustained leisure engagement (S. Lee & Park, 2021; N. Kim & Lim, 2012), making it crucial to examine whether serious leisure still provides psychological benefits under such constraints.

Contrasted with casual leisure, serious leisure requires sustained personal investment, perseverance despite constraints, and ongoing skill cultivation—qualities that may be especially beneficial for emerging adults. In this study, meaningful leisure refers specifically to Stebbins’ (1982, 2007) conception of serious leisure: systematic, committed pursuits that generate enduring satisfaction and personal growth. Unlike casual leisure, which yields short-lived hedonic pleasure, serious leisure provides deeper satisfaction and more durable psychological benefits such as self-actualization, enhanced self-image, and lasting fulfillment. These benefits, along with meaning, confidence, and social capital cultivated over time, are particularly relevance for university students facing academic pressure and identity development challenges.

Importantly, serious leisure exists under the broader umbrella of “serious pursuits,” which also includes devotee work—occupational activities pursued with the same passion, skill development, and intrinsic rewards as serious leisure (Elkington & Stebbins, 2014). This broader perspective encompasses socially valued and intellectually enriching pursuits, such as scholé-like experiences emphasizing contemplation, self-improvement, or civic contribution. Such activities may broaden students’ thought–action repertoires and strengthen positive emotions in ways consistent with the broaden-and-build theory. Prior studies demonstrate that serious leisure promotes elevated positive emotion and cognition (Kelly et al., 2019; Chen et al., 2020), which are foundational to the development of PsyCap. Nonetheless, empirical understanding of how these processes unfold within Asian higher education contexts remain limited, highlighting the need for studies examining culturally specific mechanisms.

Drawing on the broaden-and-build theory, this study addresses these gaps by testing a sequential mediation model in which serious leisure promotes positive emotion, which in turn fosters positive thought, enhances PsyCap, and contributes to life satisfaction (see Figure 1). The specific research hypotheses propose that participation in serious leisure is positively associated with life satisfaction among Korean university students, and that this association is sequentially mediated by positive emotion, positive thought, and PsyCap. By elucidating these pathways, this study aims to advance theoretical understanding of leisure-well-being mechanisms and provide culturally relevant insights for interventions designed to support student well-being in Korea.

## 2. Materials and Methods

### 2.1. Participants

Participants were 480 Korean university students (47.5% male) recruited through EMBRAIN, the largest online research agency in Korea, known for its demographically representative panels. EMBRAIN maintains a panel of approximately 1.78 million registered members in Korea, managed based on demographic variables (e.g., age, gender, region) and lifestyle information, while adhering to strict regulations to maintain data quality. Eligible participants were full-time students enrolled in Korean universities, with no additional inclusion criteria beyond willingness to participate.

During the sampling process, individuals who reported no prior engagement in serious leisure activities were excluded through the following procedure. First, all potential participants who expressed interest in the study were presented with the concepts and examples of serious leisure and casual leisure. Next, they were asked to identify one representative leisure activity in which they had been regularly and periodically participating within the past 6 months. Following this, inquiries were made regarding the extent of their participation in that activity (duration, frequency, and intensity). Participants who reported no experience in serious leisure activities were subsequently excluded during the data collection phase by EMBRAIN, and data were collected only from the remaining eligible respondents. This exclusion was implemented because the serious leisure measure is designed and validated for individuals who actively participate in serious leisure activities; therefore, including non-participants would be conceptually inappropriate and could introduce measurement error or distort the factor structure due to random or uninformed responses. Given that the purpose of this study was to examine how serious leisure influences positive emotion and psychological capital and ultimately life satisfaction, restricting the sample to active serious leisure participants was methodologically justified and aligned with the theoretical framework.

The research team requested EMBRAIN to conduct stratified random sampling targeting university students based on allocation criteria for gender and region, aligned with South Korea’s population demographics per Statistics Korea guidelines, to ensure representativeness. The sample included 90 (18.8%) freshmen, 120 (25.0%) sophomores, 120 (25.0%) juniors, and 150 (31.3%) seniors, with ages ranging from 18 to 28 years (M = 21.88, SD = 2.14). Participants received small monetary compensation for completing the survey, as per EMBRAIN’s standard practice.

### 2.2. Procedures

Prior to the main survey, a pilot test was conducted with 20 university students to assess the face and content validity of the questionnaire items. Participants were asked to provide feedback on item clarity, wording appropriateness, and ease of understanding. Based on their feedback, minor revisions were made to improve item readability. Additionally, four faculty experts in leisure social psychology, sport sociology, and psychology evaluated the clarity and representativeness of the items in relation to the constructs. The item-level content validity index (I-CVI) ranged from 0.80 to 1.00, indicating satisfactory content validity (items rated on a 4-point scale; 1 = not relevant, 4 = highly relevant).

Qualified panel members were then sent invitation emails and links to participate in an online self-administered survey. Prior to participation, an informed consent form was presented, outlining the study purpose, voluntary participation, and guarantees of data confidentiality and anonymity; only respondents who agreed and completed the survey were included in the final analysis. After receiving approval from the Institutional Review Board of Konyang University (KYU 2021-08-033-001), data were collected in accordance with the Declaration of Helsinki. The web-based survey, conducted from January to February 2023, collected data on serious leisure participation, PsyCap, and life satisfaction, requiring approximately 15 min to complete.

Response data were reviewed for quality by identifying careless or invalid responses, such as excessively short response times (completion in under 10 min for the entire survey) or repeated answer patterns (straightlining: selecting the same response option for 80% or more of consecutive items within a scale). Cases that failed to meet the criteria or contained excessive missing values (more than 10% of the total items) were excluded, resulting in a final valid sample of N = 480.

### 2.3. Measures

*Serious leisure*. Serious leisure was measured using the short-form scale developed by Ko and Kim (2016), which extracted one representative single item from each sub-dimension of M. Kim’s (2009) Serious Leisure Scale (SLS), originally derived from Gould et al.’s (2008) Serious Leisure Inventory and Measure (SLIM). This unidimensional abbreviated version was selected to minimize respondent burden while preserving validity, consistent with evidence from prior short-form adaptations (e.g., Özdemir et al., 2020; Cronbach’s α > 0.80). Each item represents one of Stebbins’ (1982) six defining qualities—perseverance, leisure career, personal effort, durable benefits, unique ethos, and leisure identity—and has demonstrated cultural relevance for Korean college students, for whom long scales may increase fatigue and response bias due to academic pressure and collectivistic norms.

This scale has been successfully applied in subsequent Korean leisure research (e.g., M. Kim et al., 2022; Ko, 2017), demonstrating strong internal consistency across studies. In M. Kim et al.’s (2022) study, the scale showed excellent reliability (Cronbach’s α = 0.903), while Ko (2017) reported good reliability (Cronbach’s α = 0.840). In the present study, all items were rated on a 5-point Likert scale (1 = not at all, 5 = a great deal), and higher scores indicate higher perceived serious leisure experience. Prior to the survey, participants were provided with definitions and examples distinguishing serious leisure (e.g., dedicated sports, volunteer work) from casual leisure (e.g., watching TV, informal socializing) and were instructed to respond based on their own serious leisure activities, excluding academic- or job-related training. The scale demonstrated good reliability (Cronbach’s α = 0.873) and convergent validity (AVE = 0.540) in this study.

*Positive emotion***.** In this study, only the positive emotion subscale from the Short-Form Positive and Negative Affect Schedule (SF-PANAS; Kercher, 1992) was used. This subscale consists of five items rated on a 5-point Likert scale (1 = not at all, 5 = a great deal), assessing the frequency of specific positive emotions experienced during the past seven days (e.g., inspired, excited). Internal consistency was acceptable (α = 0.82). Higher scores reflect greater levels of positive emotion. The decision to use only the positive emotion items was conceptually grounded in Fredrickson’s (2001) broaden-and-build theory, which emphasizes the role of positive emotions in supporting the development of cognitive resources.

*Positive thought.* Positive thoughts were measured using the Automatic Thought Questionnaire-Positive (ATQ-P), originally developed by Ingram and Wisnicki (1988), refined by S. Kim (2016) and standardized for Koran samples by J. Lee and Kim (2002). This 24 items scale comprises four subscales: positive daily functioning (6 items; α = 0.89), exemplified by the item “I am comfortable with life.”; positive self-evaluation (9 items; α = 0.91), as in “I take good care of myself.”; others’ evaluations of self (6 items; α = 0.88), as reflected in “I take good care of myself”; and positive future expectations (2 items; α = 0.87), illustrated by “My future looks bright.” Participants rated the frequency with which they experienced each thought over the past week using a 5-point Likert scale (1 = never, 5 = always). Higher scores on each subscale reflect more frequent positive thoughts.

*Positive psychological capital.* PsyCap was measured using the Korean version of the Positive Psychological Capital Scale (K-PPC), originally developed by Luthans et al. (2007) and standardized for Korean samples by B. Kim (2015). This scale comprises 18 items rated on a 5-point Likert scale (1 = not at all, 5 = a great deal) and is divided into four subscales: self-efficacy (5 items; α = 0.92), optimism (5 items; α = 0.85), hope (5 items; α = 0.87), and resilience (3 items; α = = 0.88). Representative items were included “I feel confident helping to set target/goals in my area of work” (self-efficacy), “I usually manage difficulties one way or another at work” (resilience), “I am optimistic about what will happen to me in the future as it pertains to my studies” (optimism), and “I can think of many ways to reach my current goals regarding my studies” (hope). Higher scores reflect greater levels of PsyCap.

*Life Satisfaction*. Life satisfaction was measured using the Satisfaction with Life Scale (SWLS; Diener et al., 1985), which consists of five items rated on a 7-point Likert scale (1 = strongly disagree, 7 = strongly agree). In this study, the scale demonstrated acceptable internal consistency (α = 0.87). A representative item is “In most ways my life is close to my ideal.” Higher scores reflect greater levels of life satisfaction.

### 2.4. Statistical Analysis

First, SPSS Version 25.0 was used to generate descriptive statistics and reliability analyses for all scales. Then, Structural Equation Modeling (SEM) was conducted using AMOS 21.0 to examine the structural relationships in the proposed model, and indirect effects were tested via PROCESS Macro (version 3.5) with bootstrapping. SEM included two steps. The first was to testify the validity of a measurement model through a confirmatory factor analysis by AMOS 21. Model fit was examined using Comparative Fit Index (CFI), the Tucker–Lewis Index (TLI) and Root Mean Square Error of Approximation (RMSEA), as they are less affected by sample size and represent model parsimony (Hu & Bentler, 1999). Values over 0.90 for CFI and TLI, and values lower than 0.08 for RMSEA, were used to determine a good-fit model.

To further assess discriminant validity among the constructs, the Heterotrait–Monotrait (HTMT) ratio of correlations was calculated using the observed indicators (Henseler et al., 2015). HTMT values below 0.85 indicate sufficient distinction between constructs. As shown in Table 1, all ratios were below this threshold (ranging from 0.28 to 0.76), confirming discriminant validity for serious leisure, positive emotion, positive capital, positive thought, and life satisfaction.

**Figure 2:**
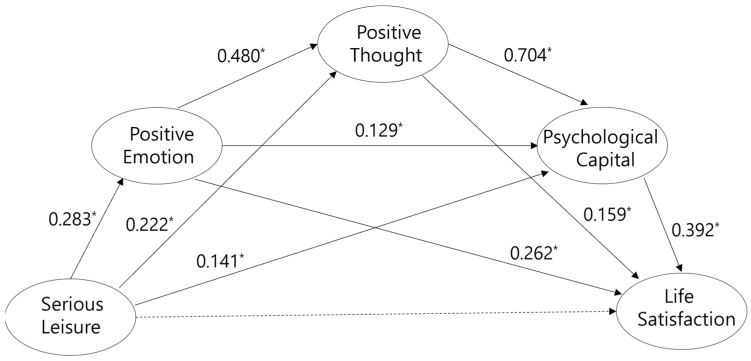
Final structural model with standardized path coefficients. Solid lines represent statistically significant paths (* *p* < 0.05); whereas dotted lines indicate non-significant paths.

In the second step, the hypothesized structural model (see Figure 2) was tested using PROCESS Macro 3.5, with the significance of indirect paths assessed via bootstrapping.

## 3. Results

### 3.1. Descriptive Statistics for the Study Variables

The means, standard deviations, skewness, kurtosis and Pearson correlations of variables in this study are listed in Table 2. Composite variables were computed using mean scores for each scale rather than true factor scores. All variables showed acceptable levels of normality, with absolute skewness values below 3.0 and kurtosis values below 10.0. Mardia’s multivariate kurtosis was 2211.16 with a normalized estimate of 65.44, suggesting that the data significantly departed from multivariate normality (*p* < 0.001). In response, bootstrapped standard errors were applied using 5000 resamples with the bias-corrected percentile method to obtain robust parameter estimates in both the CFA and structural model analyses. There was a significant positive correlation between serious leisure and positive emotion (*r* = 0.24, *p* < 0.05), positive thought (*r* = 0.32, *p* < 0.05), PsyCap (*r* = 0.39, *p* < 0.05) and life satisfaction (*r* = 0.31, *p* < 0.05). There was also a significant positive correlation between life satisfaction and positive emotion (*r* = 0.46, *p* < 0.05), positive thought (*r* = 0.56, *p* < 0.05) and PsyCap (*r* = 0.60, *p* < 0.05).

### 3.2. CFA for Measurement Model

The measurement model in this study was assessed using confirmatory factor analysis (CFA). As presented in Table 3, the model demonstrated an acceptable fit to the data (χ^2^ = 1523.697, *df* = 666, Q = 2.288, TLI = 0.920, CFI = 0.928, RMSEA = 0.052). To complement Cronbach’s alpha and provide a more robust reliability estimate, McDonald’s omega coefficients were additionally calculated for all constructs (ω = 0.874~0.91). During the CFA process, the original four-factor structure of the Positive Thought scale demonstrated inadequate discriminant validity, with high inter-factor correlations, cross-loadings, and several items falling below recommended loading thresholds (λ < 0.50) and AVE criteria. Therefore, only the two theoretically coherent and empirically stable subdimensions—Positive Self-Evaluation and Others’ Evaluations of Self—were retained in the final model. This refinement improved overall model fit and yielded a more reliable and conceptually meaningful construct representation. In the assessment of convergent validity, although the AVE for Serious Leisure is close to the 0.50 threshold (0.54), it is acceptable given the high Composite Reliability (CR = 0.874), indicating strong internal consistency and supporting convergent validity (Fornell & Larcker, 1981; Hair et al., 2019). Established guidelines note that AVE values of 0.40 or higher are permissible when accompanied by CR and Cronbach’s alpha scores of 0.60 or greater (Sharma et al., 2025), ensuring the construct’s reliability for subsequent analyses.

### 3.3. Hypothesized Model

The hypothesized model demonstrated an acceptable fit to the data (χ^2^ = 1563.057, *df* = 686, Q = 2.279, TLI = 0.920, CFI = 0.926, RMSEA = 0.052). Figure 2 presents the standardized parameter estimates for this model. All paths were statistically significant, with the exception ofc the path from serious leisure to life satisfaction. That is, there was no significant direct association between serious leisure and life satisfaction in the model (Table 4).

The findings from the bootstrapping analysis, which evaluated the statistical significance of the proposed indirect paths, are summarized in Table 5. Indirect effects were tested using PROCESS Macro (Model 4) with bias-corrected and accelerated (BC) bootstrapping (5000 resamples) to estimate path coefficients and 95% confidence intervals. To begin with, the pathway involving positive emotion linking serious leisure to life satisfaction (Path 1) yielded an estimate of 0.074, with a 95% confidence interval (CI) ranging from 0.028 to 0.121. As the interval does not include zero, this association can be interpreted as statistically meaningful. Consistent with this result, the indirect pathways through positive thought (Path 2: 0.156, 95% CI: [0.099, 0.214]) and PsyCap (Path 3: 0.055, 95% CI: [0.008, 0.102]) also fell within non-zero confidence intervals, supporting the robustness of these links. Furthermore, all three sequential mediation paths—Path 4 (0.096, 95% CI: [0.054, 0.137]), Path 5 (0.014, 95% CI: [0.003, 0.029]), and Path 6 (0.014, 95% CI: [0.012, 0.039])—exhibited confidence intervals entirely above zero, suggesting stable and reliable associations across these steps. Lastly, the indirect association captured by Path 7 was estimated at 0.008 (95% CI: [0.007, 0.024]), again falling outside the null range, indicating that the overall sequential process from serious leisure to life satisfaction is statistically tenable.

## 4. Discussion

This study examined how Korean university students’ voluntary participation in serious leisure is associated with positive affect and cognition, which may relate to higher levels of PsyCap and life satisfaction, thereby supporting their overall well-being. Korean students face heavy stressors, including intense academic pressures from competitive exams, societal expectations for career success, and social obligations rooted in collectivist values (Hofstede, 2011). These challenges can lead to anxiety, burnout, and reduced well-being, making effective coping mechanisms essential (S. Lee & Park, 2021). Serious leisure may function as a valuable coping strategy, offering structured opportunities that are linked to emotional regulation, stress relief, and resilience-building through sustained engagement in meaningful activities such as sports or cultural pursuits (Fredrickson, 2001). For instance, participation in a music club can foster joy and self-efficacy, counteracting academic stress, while volunteer work may enhance social connectedness and purpose- an outcome that aligns with collectivist norms emphasizing harmony, interdependence, and contribution to the group. Within this sociocultural framework, leisure participation may not only support individual well-being but also strengthen relational ties and social identity. Fredrickson’s (2001) broaden-and-build theory underpins this process, linking positive emotion derived from serious leisure to cognitive processes that build PsyCap.

This study examined associations between serious leisure participation and life satisfaction among Korean college students, testing a sequential mediating model involving positive emotions, thought, and psychological capital (PsyCap), guided by Fredrickson’s (2001) broaden-and-build theory. In modern society, leisure plays a pivotal role in enhancing college students’ quality of life and satisfaction (Hofstede, 2011). Korean students face significant stress from academic pressures and societal expectations for career success, making leisure activities critical for psychological and emotional stability. Recent studies highlight that leisure is not merely a stress-relief mechanism but also fosters positive emotions, creativity, and social bonds, contributing to holistic well-being (Fredrickson, 2001; S. Lee & Park, 2021). In this context, this study extends the leisure research literature by exploring how serious leisure relates to life satisfaction through psychological mechanisms, offering insights into its potential relevance as a psychological resource for university students. The following discussion is based on the key findings related to the study’s hypotheses.

First, serious leisure participation was significantly correlated with life satisfaction, aligning with prior research demonstrating that leisure activities reduce stress, enhance life satisfaction, and strengthen social bonds (Havitz et al., 2013; N. Kim & Lim, 2012; S. Lee & Park, 2021). Serious leisure, characterized by sustained commitment and skill development, may serve as a structured outlet that helps Korean college students manage psychological stresses associated with academic demands and societal expectations. Activities such as sports or cultural pursuits offer opportunities for self-expression and social connection—elements that resonate deeply within a collectivist culture valuing group cohesion and shared achievement (Heo et al., 2013a). At the same time, collectivist orientations may shape how students experience and express positive emotions, favoring socially engaged forms of happiness, such as belonging and harmony over individual pride or personal achievement (Kitayama et al., 2006). This cultural emphasis may amplify the social bonding and community aspects of serious leisure, thereby strengthening its direct association with PsyCap and life satisfaction. However, because many Korean students prioritize academic and career preparation, opportunities for serious leisure remain limited. This constraint underscores the need for institutional support, such as expanding sports facilities, offering liberal arts sports classes, and implementing leisure programs like campus clubs or cultural workshops. These initiatives, when designed and managed by leisure professionals such as recreation coordinators or sports coaches may help increase access to serious leisure, fostering psychological health and supporting students’ transition to adulthood as engaged, resilient members of society (Stebbins, 2020). Further research is needed to evaluate the effectiveness of such programs in promoting well-being, providing actionable insights for leisure professionals to optimize program design and implementation (S. Lee & Park, 2021).

Second, correlation analyses revealed significant associations between serious leisure and positive emotions, thought, PsyCap, and life satisfaction. In the SEM model, serious leisure did not directly predict life satisfaction but showed significant indirect associations through positive emotions, thought, and PsyCap, consistent with prior findings (K. Kim & Lee, 2019). A study of high school students reported similar indirect effects via PsyCap (Choi & Yang, 2018). These results align with Fredrickson’s broaden-and-build theory, suggesting that positive emotions broaden individuals’ thought–action repertoires, facilitating cognitive and behavioral flexibility that fosters enduring psychological resources such as PsyCap (Fredrickson, 1998; Cohn & Fredrickson, 2009). This process may be particularly salient in collectivist societies, where emotional regulation and prosocial engagement are emphasized as means of maintaining group harmony and collective well-being (Markus & Kitayama, 1991).

Third, PsyCap was significantly associated with life satisfaction, reinforcing its established role in enhancing subjective well-being (Avey et al., 2010; S. Lee & Park, 2021). PsyCap’s malleability, shaped by environmental factors like leisure, makes it a promising target for educational interventions (Luthans et al., 2010). Serious leisure fosters PsyCap sub-factors, particularly self-efficacy and resilience, by providing opportunities for skill mastery and overcoming challenges (Heo et al., 2013b; K. Kim & Lee, 2019). For example, a student engaged in a sports club may experience increased confidence through consistent practice (self-efficacy) and perseverance through setbacks (resilience), which may be associated with greater life satisfaction. Positive emotions, as noted by Fredrickson (2013), facilitate diverse and creative thinking, aligning with findings that positive affect enhances cognitive flexibility (Isen, 2008). In the Korean context, where academic stress can undermine confidence, serious leisure offers a structured pathway to bolster PsyCap, supporting students’ psychological resilience and academic engagement. In collectivist cultures, resilience and optimism may also be reinforced through social belonging and shared goals rather than purely individual achievement, suggesting that the benefits of serious leisure extend beyond personal competence to collective efficacy and relational support.

Fourth, this study confirmed serious leisure’s associations with positive emotions, thought, PsyCap, and life satisfaction, corroborating prior research (Heo et al., 2013b; Leversen et al., 2012). Positive emotions elicited during leisure activities enhance stress resilience and overall well-being, as they trigger upward spirals of emotional and cognitive growth (Fredrickson & Levenson, 1998; Fredrickson, 2004; S. Lee & Park, 2021). Serious leisure also fosters self-actualization and meaning in life, critical for Korean students navigating societal pressures (Heintzman & Mannell, 2003). Participation in cultural or volunteer activities can cultivate a sense of purpose and belonging, reflecting collectivist ideals of mutual support and social contribution. Moreover, Newman et al. (2014) found that positive leisure experiences build psychological resources, enhancing quality of life.

Fifth, a key conceptual decision in this study was to focus on the positive emotion subscale of the PANAS. This choice was theoretically grounded in Fredrickson’s broaden-and-build theory, which emphasizes how *positive* emotions—rather than the absence of negative affects—broaden cognitive and behavioral repertoires, fostering the development of enduring psychological resources such as optimism, resilience, and self-efficacy (Fredrickson, 1998, 2001, 2004). Measuring positive affect specifically allowed the study to capture emotions that reflect the broaden-and-build process most directly, such as enthusiasm, inspiration, and alertness. Moreover, because the focus was on upward emotional spirals that facilitate psychological growth rather than emotional recovery from distress, the positive affect dimension aligned more precisely with the theoretical aims. While this approach limits the ability to explore how negative emotions interact with leisure participation, it provides a clearer test of the mechanisms proposed by Fredrickson’s model in the context of serious leisure and PsyCap development.

Overall, these findings support the broaden-and-build theory as a valuable framework for understanding how leisure activities may be associated with psychological benefits, particularly in relation to PsyCap and life satisfaction among Korean college students. Importantly, the collectivist cultural context may shape both the mechanisms and expressions of these relationships, emphasizing communal engagement, emotional interdependence, and shared resilience as pathways to well-being. Future interventions should consider cultural orientations when designing leisure-based well-being programs to maximize their relevance and impact across diverse contexts.

## 5. Limitations and Future Directions

This study has several limitations that warrant consideration. First, reliance on self-report surveys may introduce response bias, highlighting the need for future research using observational or multi-informant data. Second, the cross-sectional design limits insights into the temporal dynamics linking serious leisure with PsyCap and life satisfaction; longitudinal studies are needed to clarify causal relationships. Third, although SEM was used to test theoretical pathways, the correlational nature of the data precludes definitive causal inferences. Fourth, single-timepoint data collection may inflate response consistency due to common method bias (Podsakoff et al., 2003).

Fifth, the focus on Korean university students may limit generalizability, as collectivist cultural values differ from those in more individualistic contexts (Hofstede, 2011). Cross-cultural comparisons are recommended to validate findings and enhance the generalizability of Fredrickson’s broaden-and-build theory across diverse settings. Sixth, the use of the five-item SF-PANAS subscale (Kercher, 1992) captures a narrow range of restricted range of positive emotions, potentially omitting affective states relevant to Fredrickson’s (2001) theory. This limitation suggests that future research should consider expanding the number or breadth of positive emotion indicators to better capture the full affective range described in broaden-and-build theory. Finally, potential confounding variables-such as socioeconomic status or type of educational institution-may have influenced the results; controlling for these factors would strengthen the robustness of future findings.

## 6. Conclusions

### 6.1. Theoretical Contributions and Implications

Despite existing studies, research on leisure, positive emotions, positive PsyCap, and life satisfaction remains limited, particularly regarding their structural relationships. This study’s findings highlight the associations of serious leisure participation and PsyCap, suggesting implications for fostering societal well-being. Using a Korean undergraduate sample, this study supported Fredrickson’s (1998, 2001) broaden-and-build theory, demonstrating correlations between serious leisure and life satisfaction in a non-Western context. As serious leisure participation correlates with enhanced mental health, future research should explore additional factors and pathways to increased well-being.

In line with Stebbins’s conceptualization of serious leisure as a potential “career” (Stebbins, 2001), younger individuals may experiment with various activities to find a good fit between their personality and *habitus* (Bourdieu, 1977) and the demands of those activities. This developmental exploration implies that long-term participation in serious leisure may serve as a resource that buffers stress in later adulthood (Brown et al., 2008), contributes to sustained well-being across the lifespan (Heo et al., 2013b) and potentially supports optimal aging or adjustment in retirement (Liechty et al., 2017). Future research should further investigate these long-term pathways and mechanisms through which serious leisure fosters resilience and life satisfaction over time.

### 6.2. Implications for Practitioners

The results indicate that serious leisure is a valuable resource for college students, correlating with psychological health amidst the pressures of transitioning to adulthood. Participation in serious leisure was associated with higher levels of PsyCap sub-factors (self-efficacy, optimism, hope, resilience), which in turn were positively correlated with life satisfaction. These results underscore the importance of promoting campus-based leisure programs, such as sports clubs or cultural workshops, developed by recreation professionals to encourage student engagement in meaningful leisure pursuits.

At the same time, it is important to acknowledge that serious leisure can have potential downsides. Its intensive nature may, for some individuals, lead to excessive involvement or even forms of behavioral addiction, and it can reduce the time available for academic work, family relationships, paid employment, or other types of leisure (see, e.g., Elkington & Stebbins, 2014). Recognizing both the benefits and risks of serious leisure participation can help practitioners and university administrators design programs that promote balanced engagement—encouraging students to develop healthy, sustainable leisure “careers” that enhance well-being not only during college years but also throughout later stages of life.

## Figures and Tables

**Table 1:** HTMT Matrix for Discriminant Validity.

Variables	1	2	3	4	5
1. serious leisure	-	0.28	0.35	0.44	0.36
2. positive emotion	0.28	-	0.50	0.47	0.51
3. positive thought	0.35	0.50	-	0.76	0.68
4. PsyCap	0.44	0.47	0.76	-	−0.69
5. life satisfaction	0.36	0.51	0.68	0.69	

**Table 2:** Descriptive statistics and correlations for the study variables.

Variables	1	2	3	4	5
1. serious leisure (M = 3.226, SD = 0.786)	1				
2. positive emotion (M = 2.423, SD = 0.769)	0.235 *	1			
3. positive thought (M = 2.926, SD = 0.763)	0.323 *	0.420 *	1		
4. PsyCap (M = 3.247, SD = 0.661)	0.393 *	0.430 *	0.730 *	1	
5. life satisfaction (M = 3.699, SD = 1.128)	0.308 *	0.461 *	0.555 *	0.602 *	1
Skewness	−0.303	0.081	−0.185	−0.342	−0.091
Kurtosis	0.233	−0.514	−0.480	0.594	−0.203

Note. * *p* < 0.05.

**Table 3:** CFA for measurement model.

2nd Order Factor	1st Order Factor	Item	*λ*	AVE	CR
	Serious Leisure	sl1	0.595	0.540	0.874
		sl2	0.619		
		sl3	0.774		
		sl4	0.806		
		sl5	0.795		
		sl6	0.789		
	Positive Emotion	pe1	0.817	0.502	0.832
		pe2	0.530		
		pe3	0.661		
		pe4	0.629		
		pe5	0.771		
positive thought	Positive self-evaluation	sel1	0.838	0.750	0.900
		sel2	0.866		
		se3	0.893		
	others evaluations of self	oe1	0.849	0.808	0.894
		oe2	0.940		
PsyCap	Efficacy	e1	0.825	0.681	0.914
		e2	0.882		
		e3	0.853		
		e4	0.807		
		e5	0.754		
	Optimism	o1	0.826	0.536	0.849
		o2	0.696		
		o3	0.815		
		o4	0.663		
		o5	0.594		
	Hope	h1	0.775	0.588	0.877
		h2	0.842		
		h3	0.733		
		h4	0.748		
		h5	0.730		
	Resilience	r1	0.823	0.707	0.878
		r2	0.831		
		r3	0.868		
	life satisfaction	ls1	0.701	0.562	0.865
		ls2	0.765		
		ls3	0.822		
		ls4	0.710		
		ls5	0.746		

**Table 4:** Direct effects in the final model.

Path	*B*	S.E.	*t* Value
serious leisure → positive emotion	0.283 *	0.051	5.304
serious leisure → positive thought	0.222 *	0.037	8.553
serious leisure → PsyCap	0.141 *	0.028	3.724
serious leisure → life satisfaction	0.043	0.054	0.963
positive emotion → positive thought	0.480 *	0.045	8.553
positive emotion → PsyCap	0.129 *	0.036	2.823
positive emotion → life satisfaction	0.262 *	0.069	4.889
positive thought → PsyCap	0.704 *	0.062	10.892
positive thought → life satisfaction	0.159 *	0.133	1.888
PsyCap → life satisfaction	0.392 *	0.151	4.286

Note. * *p* < 0.05.

**Table 5:** Mediating effects in the final model.

Path			95% C.I.	
	*B*	S.E.	LLCI	ULCI
(1) serious leisure → positive emotion → life satisfaction	0.074	0.024	0.028	0.121
(2) serious leisure → positive thought → life satisfaction	0.156	0.029	0.099	0.214
(3) serious leisure → PsyCap → life satisfaction	0.055	0.024	0.008	0.102
(4) serious leisure → positive emotion → positive thought → life satisfaction	0.096	0.021	0.054	0.137
(5) serious leisure → positive emotion → PsyCap → life satisfaction	0.014	0.007	0.003	0.029
(6) serious leisure → positive thought → PsyCap → life satisfaction	0.014	0.013	0.012	0.039
(7) serious leisure → positive emotion → positive thought → PsyCap → life satisfaction	0.008	0.008	0.007	0.024

## Data Availability

Data are available upon request.
